# Immunity in digestive diseases: new drugs for inflammatory bowel disease treatment—insights from Phase II and III trials

**DOI:** 10.1007/s00535-024-02130-x

**Published:** 2024-07-09

**Authors:** Sara Massironi, Federica Furfaro, Sarah Bencardino, Mariangela Allocca, Silvio Danese

**Affiliations:** 1 Division of Gastroenterology, Fondazione IRCCS San Gerardo dei Tintori, Via Pergolesi 3, Monza, Italy; 2 Gastroenterology and Endoscopy, IRCCS San Raffaele Hospital, Milan, Italy; 3 Vita-Salute San Raffaele University, Milan, Italy

**Keywords:** Inflammatory bowel disease, Crohn's disease, Ulcerative colitis, S1P modulators, IL-23 inhibitors

## Abstract

**Background:**

Inflammatory bowel disease (IBD), encompassing Crohn's disease (CD) and ulcerative colitis (UC), continues to challenge treatment paradigms. Advancements in therapeutic options have been have been driven by Phase 2 and 3 clinical trials of new drug classes, particularly sphingosine-1-phosphate (S1P) modulators and interleukin-23 (IL-23) inhibitors.

**Methods:**

This review synthesizes findings from Phase 2 and 3 clinical trials conducted up to early 2024, focusing on the impact of S1P modulators and IL-23 inhibitors on IBD management. Drugs such as ozanimod, etrasimod, risankizumab, mirikizumab, guselkumab, and brasikumab were evaluated for their efficacy and safety profiles.

**Results:**

S1P modulators, such as ozanimod and etrasimod, effectively regulate immune cell trafficking to reduce inflammation and several trials highlight their clinical effectiveness in both inducing and maintaining remission in IBD, highlighting its long-term safety and sustained therapeutic effects. Additionally, IL-23 inhibitors including risankizumab, mirikizumab, and guselkumab, which disrupt key inflammatory cytokine pathways, have already shown significant effectiveness in inducing and maintaining remission in both CD and UC, with favorable safety profiles across multiple studies, suggesting their potential as critical components in managing IBD.

**Conclusions:**

The clinical trials indicate that both S1P modulators and IL-23 inhibitors offer promising therapeutic benefits and maintain strong safety profiles, positioning them as potential cornerstone treatments for IBD. Despite these advancements, further exploration into long-term safety and the development of personalized treatment strategies is essential for maximizing clinical outcomes.

## Introduction

The relationship between the immune system and gastrointestinal health is central to the understanding and treatment of inflammatory bowel disease (IBD). This group of diseases, including Crohn's disease (CD) and ulcerative colitis (UC), is characterized by chronic inflammation in the digestive tract and its prevalence is increasing worldwide [1]. The cause is multifactorial and includes genetic predispositions, environmental factors, and an abnormal immune response (2,3).

Recent advances in immunology have enabled new treatment approaches for IBD in early 2024 [2–14]. Traditional options include anti-tumor necrosis factor (TNF), anti-integrin [15, 16], interleukin (IL) 12/23 inhibitors [17–19], and Janus kinase (JAK) inhibitors [20–23]. Despite these options, many patients do not respond well or eventually lose their efficacy. Therefore, research into new pathways is critical to finding more effective treatments [24, 25].

**Fig. 1:**
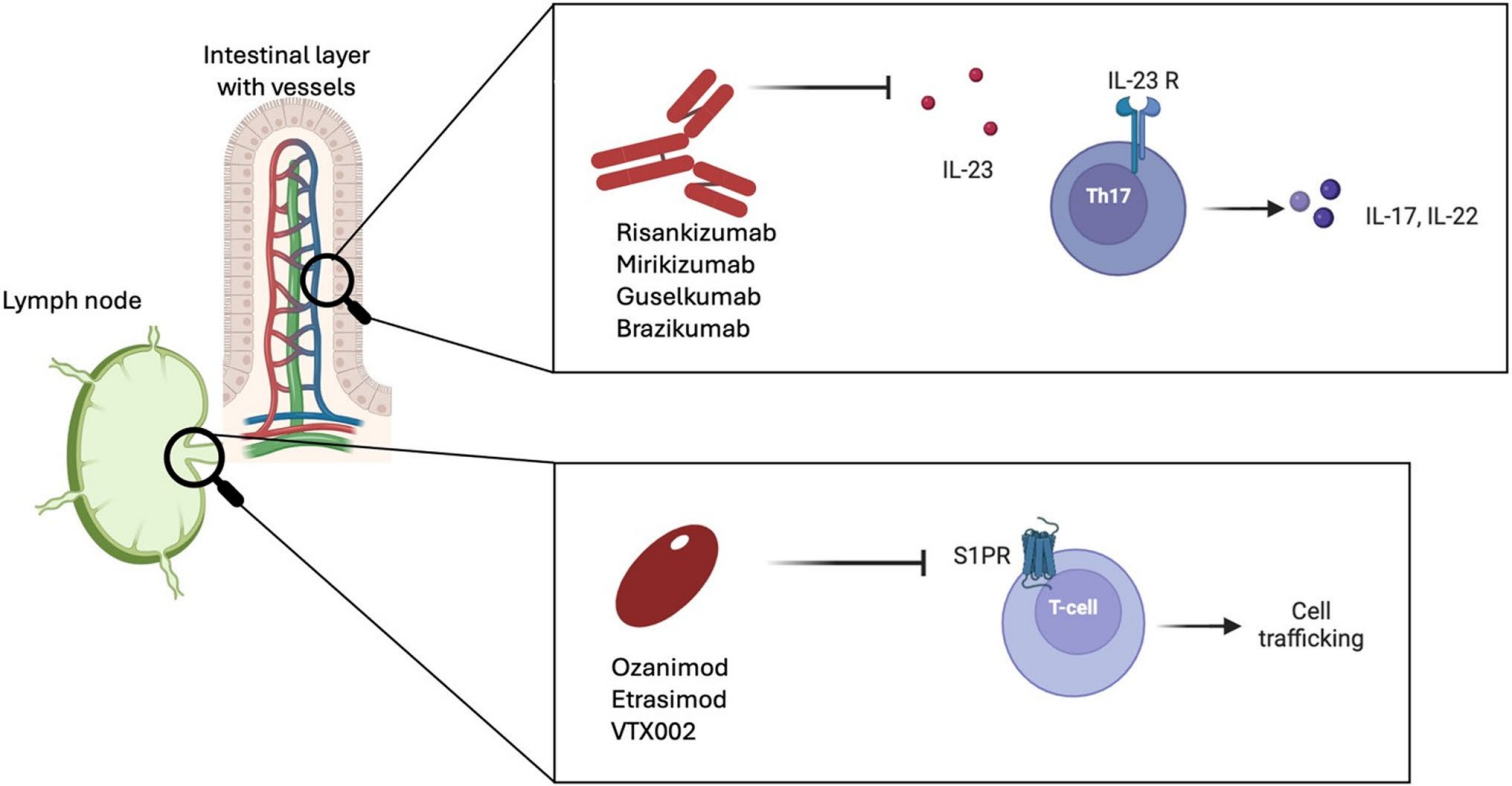
Mechanism of action of anti-IL-23 and S1P inhibitors. The inhibition of S1PR affects the exit of lymphocytes from lymphoid tissue, while the blockage of IL-23 limits the differentiation and survival of T helper 17 cells

Two promising classes of drugs are sphingosine-1-phosphate (S1P) modulators [26–31] and interleukin-23 (IL-23) inhibitors [32–37]. S1P modulators, such as ozanimod and etrasimod, are gaining attention due to their unique mechanism of action involving the regulation of immune cell trafficking [3, 38]. By affecting the exit of lymphocytes from lymphoid tissue, these agents can attenuate the inflammatory cascade underlying IBD [28]. IL-23 inhibitors, which target IL-23, a cytokine critical for the differentiation and survival of T helper 17 cells [32–34], offer a novel approach to modulate the immune response in IBD [17], potentially offering a more targeted and effective treatment option [17] (Fig. 1).

Given the complicated mechanisms of these new therapies, the future of IBD treatment lies in understanding the immune mechanisms that drive these diseases [14, 39–42].

## Drugs targeting sphingosine-1-phosphate (S1P)

Recent research has highlighted the therapeutic potential of S1P, a lipid mediator closely associated with immune cell dynamics and inflammation [43, 44].

S1P receptors (S1PR) are found on various immune cells. The biological effects of S1P are mediated via five specific G-protein-coupled receptors, S1PR1–5 [45]. Dysregulation of S1P signaling in IBD leads to increased S1P levels in the inflamed mucosa, which promotes immune cell recruitment and leads to persistent inflammation and tissue damage [46].

Drugs targeting S1PR1–5 can modulate the egress of lymphocytes from the lymph nodes, reducing their availability to contribute to inflammation in the gut. In addition, modulation of S1P signaling may help maintain the integrity of the intestinal barrier. Ozanimod and etrasimod, both S1P modulators targeting the S1P signaling pathway, have shown promising results in studies and could, therefore, be considered as a new therapeutic target [47–49]. Ozanimod targets the S1PR1 and S1PR5 and effectively reduces the migration of pathogenic immune cells into the inflamed areas of the gut. Ozanimod was first studied in multiple sclerosis and has received international approval for the treatment of relapsing forms of the disease [50]. Ozanimod has already been approved by the Food and Drug Administration (FDA) and the European Medical Agency (EMA), while approval is still pending for etrasimod, another selective S1PR modulator being studied for UC and CD, and for VTX002. Oral administration, speed, and a reliable safety profile are the main advantages of this class of drugs [26].

### Ozanimod

#### Ozanimod in UC

The introduction of ozanimod, a first-in-class S1P modulator, has significantly improved the treatment of UC [31, 51]. Its efficacy in the treatment of UC has been confirmed in several trials (Table 1) and observational studies (Table 2).

**Table 1:** Phase-2,-3-and -4 studies on ozanimod in ulcerative colitis (UC)

NCT number	Study acronym	Type of study	Aim	Study population	Primary outcome measures	Sponsor	Phases	Study status
NCT01647516	TOUCHSTONE	A Phase 2, Multicenter, Randomized, Double-Blind, Placebo-Controlled Parallel-Group Study	To evaluate the Efficacy and Safety of Ozanimod as Induction Therapy	Moderate to Severe UC (18–73 Years)	Percentage of Participants Who Achieved Clinical Remission, defined as a Mayo Score (MS) of ≤ 2 points and with no individual subscore ≥ 1 point (Central Read) At Week 8 and 32	Celgene	Phase-2	COMPLETED (2019-08-30)
NCT02435992	TRUE NORTH	A Phase 3, Multicenter, Randomized, Double-blind, Placebo-controlled Trial	To evaluate the Efficacy and Safety Study of Ozanimod as Induction and Maintenance Therapy	Moderate to Severe UC (18–75 Years)	Percentage of participants that are in Clinical remission At week 10 and 52	Celgene	Phase-3	COMPLETED (2020-06-17)
NCT02531126	TOUCHSTONE OLE	A Phase 3, Multicenter, Open-Label Extension Trial	An Extension Study of RPC1063 To evaluate the Safety and Efficacy of Ozanimod	Moderate to Severe UC (18–75 Years)	Number of Participants with Treatment-Emergent Adverse Events (TEAEs), with Adverse Events (AEs), with Serious Adverse Events (SAEs), with TEAEs Leading to Discontinuation of the Drug, with AEs of Special Interest From enrollment until at least 90 days after completion of study treatment	Celgene	Phase-3	ACTIVE, NOT RECRUITING Completion Date (estimated) 2025-02-27
NCT03915769	NA	A Phase 2/3, Multicenter, Randomized, Double-blind, Placebo-controlled Study	To Evaluate the Efficacy and Long-term Safety of Ozanimod	Japanese Subjects With Moderate to Severe UC (18–75 Years)	The proportion of subjects with clinical response, defined as a reduction from baseline in the complete MS of ≥ 3 points and ≥ 30%, and a reduction from baseline in the rectal bleeding subscore of ≥ 1 point or an absolute rectal bleeding subscore ≤ 1 point At Week 12	Celgene	Phase-3	ACTIVE, NOT RECRUITING Completion Date (estimated) 2025-03-13
NCT05644665	NA	A Phase 3, Multicenter, Randomized, Double-blind, Placebo-controlled Study	To Evaluate the Efficacy and Long-term Safety of Oral Ozanimod	Chinese Participants With Moderately to Severely Active UC (18–75 Years)	The proportion of participants with clinical remission based on MS At week 10 Maintenance Period at Week 52	Bristol-Myers Squibb	Phase-3	RECRUITING Completion Date (estimated) 2027-04-14
NCT05076175	NA	A Phase 2/3, Multicenter, Randomized, Double-Blind Study	To Evaluate the Efficacy, Safety, Pharmacokinetics and Pharmacodynamics of Oral Ozanimod (High and low doses)	Pediatric Subjects With Moderate to Severe UC With an Inadequate Response to Conventional Therapy (2–17 Years)	The proportion of participants who achieve clinical remission At Week 10 and 52	Bristol-Myers Squibb	Phase-2/ Phase-3	RECRUITING Completion Date (estimated) 2031-08-14
NCT05369832	NA	A Phase 4, Prospective, Open-label Study of Ozanimod	To investigate the Safety, Efficacy, Quality of Life, and Biomarker Response	Patients with Moderate to Severe UC treated in clinical practice(18 Years and older)	Clinical response as measured by modified MS at Week 12, Cohort 1 and 2 Up to approximately 26 weeks	Bristol-Myers Squibb	Phase-4	RECRUITING Completion Date (estimated) 2027-04-28

**Table 2:** Ongoing observational studies on Ozanimod in ulcerative colitis (UC)

NCT number	Study acronym	Type of study	Aim	Study population	Primary outcome measures	Sponsor	Study status
NCT06126835	NA	A Retrospective Observational Cohort Study	To Evaluate the Safety of Ozanimod Exposure During Pregnancy in Women With UC and Their Infants	Pregnant Women With UC and Their Offspring (18–49 Years)	Prevalence of major congenital malformations among infants born to pregnant women with UC who were exposed to ozanimod during the first trimester relative to the prevalence among (i) infants born to women with UC exposed to conventional UC treatments and (ii) infants born to women with UC exposed to advanced UC treatment during first trimester of pregnancy. Up to 10 years	Bristol-Myers Squibb	NOT YET RECRUITING Completion Date (estimated) 2031-05-31
NCT05953402	NA	A Prospective, Observational Study (Patient Registry)	To evaluate the Safety of Ozanimod Exposure in Pregnant Women With UC and Their Offspring	Pregnant Women With UC and Their Offspring (Child, Adult, Older Adult)	Event rate of Major Congenital Malformations Up to 12 months	Bristol-Myers Squibb	NOT YET RECRUITING Completion Date (estimated) 2032-06-30
NCT05382715	COLIBRI	A Four Year, Multicenter, Prospective, Observational Study in Clinical Routine	A Study to Evaluate the Utilization, Effectiveness of Ozanimod, and Quality of Life	Ozanimod-treated Participants With moderate-to-severe UC (18 Years and older)	Number of participants with Clinical Remission, defined by a partial MS of 1 plus an RBS = 0 At weeks 10, 52, and one year	Bristol-Myers Squibb	RECRUITING Completion Date (estimated) 2026-11-30

*MS* Mayo score, *TEAEs* Treatment-emergent adverse events, *AEs* Adverse events, *SAEs* Serious adverse events

The initial study of ozanimod on 88 healthy volunteers showed the drug was well tolerated. No serious adverse events (AEs) or dose-limiting toxicities were observed, and only a dose-dependent cardiac chronotropic effect was observed after the first dose [52]. A phase 2 trial (TOUCHSTONE study), which involved 197 adults with moderate to severe UC, investigated the efficacy and safety of ozanimod [53]. In this study, patients received either 0.5 mg or 1 mg of ozanimod or a placebo daily for up to 32 weeks. A higher clinical remission rate was observed for the 1 mg dose compared to placebo at both week 8 (57%) and week 32 (51%) [53]. The clinical efficacy of ozanimod in inducing remission in UC was also demonstrated in the phase 3 study (TRUE NORTH STUDY) 54. In this study, 645 patients received ozanimod hydrochloride 1 mg or placebo once daily in a double-blind manner during the induction phase (cohort 1) and 367 patients received open-label ozanimod at the same daily dose (cohort 2). The percentage of clinical remission was significantly higher in patients receiving ozanimod than in those receiving placebo, both during the induction phase (18.4% vs. 6.0%, *P* < 0.001) and during maintenance therapy (37.0% vs. 18.5%, *P* < 0.001). The percentage of clinical response was also significantly higher with ozanimod than with placebo during induction (47.8% vs. 25.9%, *P* < 0.001) and maintenance therapy (60.0% vs. 41.0%, *P* < 0.001). By week 10, the percentage of patients with histologic remission was 10.8 percentage points higher with ozanimod than with placebo, along with a reduction in Mayo score (MS) for rectal bleeding and improvements in endoscopic appearance and mucosal healing. A total of 457 patients who had responded to ozanimod during induction were re-randomized at week 52 to receive double-blind maintenance therapy with either ozanimod (*n* = 230) or placebo (*n* = 227). This remission persisted over 24 weeks of maintenance therapy, with 37.0% in the ozanimod group and 18.5% in the placebo group achieving clinical remission (difference 18.6%, *P* < 0.0001) [54]. In addition, a significantly higher proportion of patients with ozanimod achieved histologic remission. Common AEs included anemia and headache. The incidence of infections (of any severity) was similar with ozanimod during induction as with placebo and higher than with placebo during maintenance therapy. Serious infections occurred in less than 2% of patients in each group during the 52-week study. Elevated liver aminotransferase levels occurred more frequently with ozanimod [53, 54].

The long-term efficacy of ozanimod was observed in the open-label extension of the phase 2 trial (TOUCHSTONE-OLE study) 55. In this study, 170 of 197 patients receiving double-blind treatment, were enrolled in the extension phase with ≥ 4 years of follow-up. The dropout rate was 28% at year 1 and 15–18% annually through year 4. Clinical response and remission rates were 93.3% and 82.7% at week 200, respectively, with endoscopic improvement rates of 46.4% and 46.5% at weeks 56 and 104, respectively, and histologic remission rates were 46.3% and 38.5%, respectively. No new AEs were noted during the follow-up period of ≥ 4 years. These results suggest that ozanimod maintains its efficacy over a longer time [55, 56].

Long-term efficacy was demonstrated even after approximately 3 years of continuous ozanimod in UC, based on the analysis from the True North open-label extension (OLE) study [57]. This analysis involved patients with moderately to severely active UC who had responded to ozanimod after 52 weeks in the True North phase 3 study and continued treatment in the OLE for approximately 2 additional years (up to OLE week 94). The results are promising indicating sustained efficacy and a favorable safety profile over approximately 3 years of continuous ozanimod treatment. In the analysis of the observed cases, a significant percentage of patients achieved clinical response (91.4%), clinical remission (69.1%), and corticosteroid-free remission (67.9%) at OLE week 94, i.e. after a total of 146 weeks of treatment. Similarly, a significant proportion of patients achieved endoscopic improvement (73.3%), histologic remission (67.3%), and mucosal healing (56.3%) at this time point. Although efficacy rates were lower in non-responders, efficacy was maintained until OLE week 94. Regarding safety, no new signals emerged in this long-term analysis [57].

Ongoing studies continue to assess ozanimod's long-term safety and efficacy across different cohorts. NCT03915769 focuses on the long-term safety and efficacy of ozanimod in a Japanese cohort. Similarly, study NCT05644665 is designed to evaluate the efficacy and safety of ozanimod in participants with moderately to severely active UC in mainland China and Taiwan. Another study, NCT06073873, is an observational study evaluating the safety of ozanimod in real-world settings in Korean participants with moderately to severely active UC.

The effects of ozanimod on UC is also being studied in various subgroups, including pediatric patients, pregnant women, and the elderly. The NCT05076175 trial, a multicenter, randomized, double-blind, phase 2/3 study, aims to evaluate the efficacy, safety, pharmacokinetics, and pharmacodynamics of oral ozanimod in pediatric patients with moderately to severely active UC who have had an inadequate response to conventional therapy.

Additional ongoing phase 4 studies include NCT05369832, an open-label study of ozanimod in moderate to severe UC, evaluating the safety, efficacy, quality of life (QOL) impact, and biomarker response of ozanimod in participants with moderate-to-severe active UC in clinical practice, which is expected to be completed in 2027.

Observational studies on ozanimod in UC include NCT06126835, which is investigating the safety of ozanimod exposure during pregnancy, NCT05953402, a study of ozanimod in pregnant women with UC and their offspring, and NCT05382715 (COLIBRI), which is investigating the use, efficacy, and QOL of ozanimod in UC participants (Table 2).

#### Ozanimod in CD

Research into ozanimod has extended to CD, with several phase 3 trials exploring its efficacy and safety. The earlier phase-2 STEPSTONE trial, which demonstrated the benefits of ozanimod, provided the basis for further studies [58], leading to the comprehensive YELLOWSTONE clinical trial program [59], which includes two randomized, double-blind, placebo-controlled induction studies (NCT03440372 and NCT03440385), a maintenance study (NCT03464097), and an open-label extension study (NCT03467958). This program applies strict criteria to enroll patients who do not respond to or cannot tolerate at least one existing CD treatment. It uses state-of-the-art methods, such as centrally read histologic and endoscopic examinations as well as symptom monitoring using the Crohn's Disease Activity Index (CDAI). The program, with results expected between 2023 and 2026, shows a clear path for potential approval of ozanimod as a novel CD therapy.

Further studies are expanding its potential application. NCT05470985 is investigating the efficacy, safety and pharmacokinetics of the drug in pediatric patients (aged 2–17 years) with moderately to severely active CD, who have a Pediatric Crohn's Disease Activity Index (PCDAI) score ≥ 30 and a Simple Endoscopic Score for Crohn's Disease (SES-CD) ≥ 6 (or SES-CD ≥ 4 for participants with isolated ileal disease) and studying those who do not respond adequately to treatments such as corticosteroids, immunomodulators, biologics, or other systemic immunomodulatory therapies for CD. This study will help to clarify the effect of ozanimod in younger patients and is expected to be completed by 2032.

### Etrasimod

Etrasimod selectively targets S1PR (specifically S1PR1, S1PR4, and S1PR5), which play a key role in immune system regulation. By modulating these receptors, similarly to ozanimod, etrasimod effectively dampens inflammation in IBD.

#### Etrasimod in UC

The efficacy of etrasimod in treating UC has been confirmed through several clinical trials [26, 60, 61] (Table 3). The phase 2 OASIS study (NCT02447302) showed that administering etrasimod 2 mg for 12 weeks resulted in significant improvements in modified MS compared to placebo [62]. Endoscopic improvement was observed in 41.8% of patients receiving etrasimod 2 mg versus 17.8% receiving placebo (*P* = 0.003). Although some patients experienced a low-grade transient atrioventricular block, the majority of AEs were mild to moderate.

**Table 3:** Studies exploring the potential of Etrasimod in treating ulcerative colitis (UC), covering different phases, patient populations, and study statuses

NCT number	Study acronym	Type of study	Aim	Study population	Primary outcome measures	Sponsor	Phases	Study status
NCT02447302	OASIS	A Phase 2, Randomized, Double-Blind, Placebo-Controlled, Parallel Group, Multi-Center Study	To investigate Etrasimod's safety and efficacy in Moderately to Severely Active UC	Moderately to Severely Active UC, defined as per Mayo Score (MS) or Evidence of colonic UC activity on endoscopy (18–80 Years)	Clinical remission assessed by Change From Baseline in Adapted MS At Week 12	Arena Pharmaceuticals	Phase-2	Completed (Published Results)
NCT02536404	NA	Extension Study of NCT02447302	To evaluate Etrasimod's safety and efficacy in Moderately to Severely Active UC after 52 weeks	Participants with Moderately to Severely Active UC, who completed the NCT02447302 study (18–80 Years)	Number of Participants With Treatment-Emergent Adverse Events (TEAEs) and Serious Adverse Events (SAEs) Up to Week 48	Arena Pharmaceuticals	Phase-2	Completed (Published Results)
NCT04607837	GLADIATOR UC	A Randomized, Double-Blind, Placebo-Controlled, 52-Week Study	To Assess the Efficacy and Safety of Etrasimod	Subjects With Moderately Active UC, defined as a modified MS of 4 to 6 and an endoscopic score ≥ 2 and rectal bleeding score ≥ 1 (18–80 Years)	Proportion of Participants Achieving Clinical Remission At week 12 and 52 Safety up to week 56	Pfizer	Phase-2	Active, Not Recruiting
NCT05061446	NA	Dose-Ranging Study of Etrasimod	To assess the safety and efficacy of Etrasimod administered for 12 weeks, in Japanese subjects	Japanese Subjects With Moderately to Severely Active UC (18–80 Years)	Proportion of Participants Achieving Clinical Remission. At week 12 Adverse Events (AEs) up to 16 weeks	Pfizer	Phase-2	Completed (Results Not Published)
NCT03996369	ELEVATE UC 12	A Phase 3, Randomized, Double-Blind, Placebo-Controlled, 12-Week Study	To assess the Efficacy and Safety of Etrasimod	Participants with moderately to severely active UC, confirmed by endoscopy (16–80 Years)	Percentage of Participants Achieving Clinical Remission At week 12	Arena Pharmaceuticals	Phase-3	Completed (Published Results)
NCT03945188	ELEVATE UC 52	A Phase 3, Randomized, Double-Blind, Placebo-Controlled, 52-Week Study	To Assess the Efficacy and Safety of Etrasimod	Subjects with moderately to severely active UC, confirmed by endoscopy (16–80 Years)	Percentage of Participants Achieving Clinical Remission At week 12 and 52	Arena Pharmaceuticals	Phase-3	Completed (Published Results)
NCT04706793	ELEVATE UC 40 JAPAN	A Phase 3, Double-Blind, Placebo-Controlled, 40-Week Extension Study of NCT03996369	To Assess the Efficacy and Safety of Etrasimod	Japanese Subjects With Moderately to Severely Active UC (16–80 Years)	Percentage of Participants Achieving Clinical Remission At Week 40 of Study NCT03996369	Pfizer	Phase-3	Completed (Published Results)
NCT03950232	ELEVATE UC OLE	An Open-Label Extension Study	To evaluate the safety and efficacy of etrasimod	Subjects With Moderately to Severely Active UC who previously received double-blind treatment in in parent studies (16–80 Years)	Number and Severity of Safety Measures Up to approximately 8 years	Pfizer	Phase-3	Recruiting
NCT04176588	NA	A Phase 3, Randomized, Placebo-Controlled, Double-Blind, Multicenter Study	to Evaluate the Efficacy and Safety of Etrasimod for Induction and Maintenance Treatment	Subjects With Moderately to Severely Active UC, confirmed by endoscopy with ≥ 10 cm rectal involved (18–75 Years)	The proportion of Subjects With Clinical Remission Assessed by modified MS At week 12 and 40	Everstar Therapeutics Limited	Phase-3	Unknown Status
NCT05287126	NA	An Open-Label, Single-Arm Study	To Evaluate the Efficacy, Pharmacokinetics, and Safety of Etrasimod	Adolescent Subjects With Moderately to Severely Active UC (12–17 Years)	The proportion of Participants Achieving Clinical Remission as Assessed by Modified MS at Week 52. Participants who complete 52 weeks have the option to continue in a long-term extension period of up to 4 years or until marketing authorization is obtained in their country	Pfizer	Phase-2	Recruiting

*MS* Mayo score, *TEAEs* Treatment-emergent adverse events, *AEs* Adverse events, *SAEs* Serious adverse events

In the OASIS open-label extension study (NCT02536404), patients who continued treatment with etrasimod 2 mg for up to 52 weeks maintained clinical response (85%), remission (60%), and endoscopic improvement (69%). During the long-term extension study, etrasimod 2 mg showed a favorable safety profile. Despite 60% of patients experiencing AEs, most (94%) were mild or moderate, with worsening UC and anemia being the most common [63].

Another phase 2 study, NCT04607837 (GLADIATOR UC) is currently being conducted. This randomized, double-blind, placebo-controlled, 52-week study is designed to evaluate the efficacy and safety of etrasimod in patients with moderately active UC. The primary objective of this study is to determine whether oral etrasimod can be a safe and effective treatment.

Following the promising results of the phase 2 studies, further phase 3 studies, such as the ELEVATE UC 12 and ELEVATE UC 52 studies, have confirmed the efficacy and safety of etrasimod [64, 65]. Both randomized, double-blind, placebo-controlled studies enrolled adults with moderate to severe UC who had not responded to or were intolerant of previous treatments and were randomly assigned (2:1) to receive either 2 mg of etrasimod or placebo orally once daily [64, 65]. ELEVATE UC 12 independently assessed induction at week 12, while ELEVATE UC 52 included a 12-week induction phase followed by a 40-week maintenance phase with a treat-through design [65]. ELEVATE UC 12 showed that 25% of patients treated with etrasimod achieved clinical remission at week 12 compared to 15% in the placebo group (*p* = 0.026). In the ELEVATE UC 52 study, 32% of patients on etrasimod achieved clinical remission after a 12-week induction phase and a 40-week maintenance period, compared to only 7% on placebo (*p* < 0.0001). AEs were reported in 71% of the etrasimod group and 56% of the placebo group in ELEVATE UC 52 [65], while in ELEVATE UC 12, AEs affected 47% in both groups. Crucially, there were no reports of deaths or malignancies, confirming the drug's strong safety profile [64].

The ELEVATE UC OLE (Open-Label Extension) study (NCT03950232), a phase 3 study currently recruiting participants, aims to evaluate the long-term safety and efficacy of etrasimod. It includes participants with moderate to severe UC who have previously participated in double-blind, placebo-controlled phase 2 or phase 3 studies. By following patients over a longer period, this extension study will shed light on the long-term effects of etrasimod.

Other ongoing studies are focusing on specific population groups. The recently completed phase 2 study (NCT05061446) in Japan investigated etrasimod as an induction therapy. In the ELEVATE UC 40 JAPAN study (NCT04706793), participants continued treatment for 52 weeks to assess long-term efficacy. A phase 3 study (NCT04176588) is currently being conducted in China comparing etrasimod to placebo for induction and maintenance treatment in patients with moderate to severe UC.

Etrasimod is currently also being investigated in adolescents. The study NCT05287126 is an open-label, single-arm phase 2 study evaluating the efficacy, pharmacokinetics and safety of etrasimod in adolescent patients aged 12 to 17 years with moderately to severely active UC. Participants who complete the 52-week treatment can continue the study for up to four additional years as part of a long-term extension (LTE) or until marketing authorization is granted in the participant's country.

#### Etrasimod in CD

Research on etrasimod in CD is being rigorously pursued as part of the CULTIVATE clinical program (NCT04173273), a comprehensive phase 2/3 project. This multi-faceted study is designed to evaluate the efficacy, safety and tolerability of oral etrasimod as a therapy in adult participants with moderately to severely active CD who are refractory or intolerant to at least one of the current therapies for CD (i.e. corticosteroids, immunosuppressants or biologics). Sub-study A, which forms the initial phase of the CULTIVATE program, is a randomized, double-blind phase 2 study. Its primary objective is to evaluate the safety, tolerability and initial efficacy of orally administered etrasimod in participants diagnosed with moderate to severe CD. This substudy aims to determine appropriate doses for induction and maintenance therapy. Sub-study 1, a double-blind, placebo-controlled, dose-ranging induction substudy to select an induction and maintenance dose(s) of etrasimod. The results of this substudy are pivotal in selecting the optimal dose for both induction and maintenance therapy in the subsequent phase 3 analysis. Sub-study 2, a phase 3 randomized, double-blind, placebo-controlled substudy, specifically evaluates the efficacy of etrasimod in inducing a therapeutic response in CD. Sub-study 3 focuses on the maintenance phase of treatment. This substudy is a phase 3 randomized, double-blind, placebo-controlled substudy and enrolls participants who have participated in substudy 1 and substudy 2. Finally, the program extends into substudy 4, a long-term extension phase for participants who have completed at least 52 weeks of treatment under the program. This substudy is critical for evaluating the long-term effects and sustainability of etrasimod therapy in CD over time.

### Amiselimod

Amiselimod is an orally administered selective S1PR1 modulator with potentially fewer adverse effects, particularly it demonstrated a more favorable cardiac safety profile compared with other S1PR1 modulators. However, in a multicenter, randomized, double-blind, placebo-controlled, parallel-group phase IIa study, amiselimod 0.4 mg was compared with placebo over a treatment period of 14 weeks. The study showed that amiselimod 0.4 mg over 12 weeks was not superior to placebo in inducing a clinical response in CD [66]. A Phase 2, randomized, double-blind, placebo-controlled, parallel-group study to evaluate the efficacy and safety of amiselimod (MT-1303) over 12 weeks as induction therapy in patients with active mild-to-moderate UC and as maintenance therapy for up to 36 weeks, is ongoing (NCT04857112), with an expected completion date in 2024-09. The primary endpoint is the change from baseline in modified Mayo score at day 85; the secondary endpoint is the proportion of subjects with endoscopic improvement at day 85. However, results are not yet available.

## Agents targeting IL-23

IL-23) plays a pivotal role in the development of IBD [67]. As a member of the IL-12 cytokine family, it is essential for the differentiation and maintenance of T-helper 17 (Th17) cells, which produce pro-inflammatory cytokines [68]. This IL-23/Th17 axis is critical to chronic inflammation in IBD [68, 69]. Recognizing the critical role of IL-23 in disease progression, biopharmaceutical agents have been developed that neutralize IL-23 and reduce inflammation more precisely than broad-spectrum immunosuppressants [68].

The first drug of this type, ustekinumab, was originally developed for psoriasis but was approved for CD due to its efficacy. It targets the common subunit p40 of IL-12 and IL-23 and inhibits their activity. Clinical trials have shown that ustekinumab is effective in inducing and maintaining remission in patients with CD, even in patients who have not responded to previous biologics [70].

### Risankizumab and mirikizumab

Targeting specifically the p19 subunit of IL-23 these agents offer a more selective therapeutic approach [71]. Early clinical studies have shown promising results for risankizumab in both UC (Table 4) and CD (Table 5). Subsequent studies have supported the efficacy of risankizumab, suggesting that it could become an important tool in the treatment of IBD [17, 18, 72–75].

**Table 4:** Studies exploring the role of Risankizumab in ulcerative colitis (UC), covering different phases of studies, patient populations, and study statuses

NCT number	Study acronym	Type of study	Aim	Study population	Primary outcome measures	Sponsor	Phases	Study status
NCT03398148	INSPIRE	A Multicenter, Randomized, Double-Blind, Placebo-Controlled Induction Study	To evaluate the efficacy, safety, and pharmacokinetics of Risankizumab as an induction treatment and to identify the appropriate induction dose	Participants With Moderately to Severely Active UC (16–80 Years)	Participants Achieving Clinical Remission per Adapted Mayo Score (MS) At week 12	AbbVie	Phase-2 Phase-3	COMPLETED (2023-05-11) No results
NCT03398135	COMMAND	A Multicenter, Randomized, Double-Blind, Placebo-Controlled 52-week Maintenance and an Open-Label Extension Study	To evaluate the Efficacy and Safety of Risankizumab as maintenance therapy	Subjects With UC who have completed Study NCT03398148 and have achieved clinical response (16–80 Years)	Clinical Remission per Adapted MS At Week 52 Percentage of Participants with Adverse Events (AEs) Up to Week 300	AbbVie	Phase-3	ACTIVE, NOT RECRUITING Completion Date (estimated) 2028-09-25

*MS* Mayo score, *AEs* Adverse events

**Table 5:** Studies exploring the role of Risankizumab in Crohn’s Disease (CD), covering different phases of studies, patient populations, and study statuses

NCT number	Study acronym	Type of study	Aim	Study population	Primary outcome measures	Sponsor	Phases	Study status
NCT03105128 and NCT03104413	ADVANCE And MOTIVATE	Multicenter, Randomized, Double-Blind, Placebo-Controlled Induction Studies	To evaluate the Efficacy and Safety of Risankizumab as induction therapy	For ADVANCE: Subjects With Moderately to Severely Active CD (16–80 Years) For MOTIVATE: Subjects With Moderately to Severely Active CD Who Failed Prior Biologic Treatment	Clinical Remission, defined as using the average daily stool frequency (SF) ≤ 2.8 and not worse than Baseline AND average daily abdominal pain (AP) score ≤ 1 and not worse than Baseline Clinical remission (for US) as per CDAI, Endoscopic Remission as per SES-CD At Week 12	AbbVie	Phase-3	COMPLETED (2021–04-14) and (2021-05-19)
NCT03105102	FORTIFY	A Multicenter, Randomized, Double-Blind, Placebo-Controlled 52-Week Maintenance and an Open-Label Extension Study	Sub-study 1: to evaluate the efficacy and safety of Risankizumab as maintenance therapy Sub-study 2: to evaluate two different dosing regimens Sub-study 3: to evaluate the long-term safety Sub-study 4: to evaluate patient-reported outcomes, tolerability, and pharmacokinetics of risankizumab self-administered	Subjects With CD (16–80 Years)	Sub-Study 1 and 2: Percentage of Participants With Deep Remission: i.e. with CDAI Clinical Remission and with Endoscopic Response, defined as a decrease from Baseline in SES-CD At Week 52 Sub-Study 3: Number of Participants With AEs Up to Week 220 Sub-Study 4: Percentage of Participants in CDAI Clinical Remission and Percentage of Participants Rating Self-Injection Up to Week 16	AbbVie	Phase-3	ACTIVE, NOT RECRUITING Completion Date (estimated) 2026-06-09
NA	NA	Real-world Effectiveness and Safety of Risankizumab. A Belgian Multicentric Cohort Study	To evaluate the Effectiveness and Safety of Risankizumab	Patients with Moderate to Severe Multirefractory CD	Clinical remission and endoscopic response At week 24 and week 52	NA	Phase-4	COMPLETED (April 2023)
NA	NA	Risankizumab Effectiveness and Safety in Crohn’s Disease: Real-world Data From a Large Tertiary Center	To evaluate the effectiveness of Risankizumab	Patients with CD with active luminal disease, defined as HBI = 5, and/or evidence of active disease per ileocolonoscopy, and/or evidence of active disease per imaging, and/or FCP 250	Reduction of Disease activity score, steroid usage Percentage of AEs	NA	Phase-4	COMPLETED
NA	NA	Effectiveness and safety of Risankizumab induction therapy for 100 patients with Crohn's disease: A GETAID multicentre cohort study	To Evaluate the Effectiveness and safety of Risankizumab as induction therapy	Patients with CD	Steroid-free clinical remission at week 12 (HBI < 5)	NA	Phase-4	COMPLETED (May 2022)
NCT05841537	APPRISE	A Prospective, Post-marketing, Non-interventional Study of Risankizumab Evaluating Real-world Clinical Effectiveness in Crohn's Disease	To evaluate safety and effectiveness of Risankizumab in real-world	Patients with moderately to severely active CD 15 Years and older	Percentage of Participants with Clinical Remission among Participants with Clinical Response at Month 3 [Time Frame: At Month 12]	AbbVie	Observational	RECRUITING Completion Date (estimated) 2027–10-31

*SES-CD* Simple endoscopic score for Crohn's disease, *CDAI* Crohn's disease activity Index, *FCP* Fecal calprotectin, *Hs-CRP* High-sensitive C-reactive protein, *AE* Adverse events, *HBI* Harvey–Bradshaw index, *SF* Stool frequency, *AP* Abdominal pain

#### Risankizumab in UC

The INSPIRE study, a double-blind, placebo-controlled phase 3 study, evaluated the efficacy and safety of risankizumab in patients with UC who had not responded well to conventional or advanced therapies [76]. Excluding those previously treated with ustekinumab or other IL-23 inhibitors, the study randomized 975 patients 2:1 to receive either placebo or 1200 mg of risankizumab intravenously at weeks 0, 4, 8 and 12. Risankizumab achieved significantly higher clinical remission rate at week 12 compared to placebo (20.3% vs. 6.2%, *P* < 0.00001). The most common AEs included COVID-19 infections, anemia, and worsening of UC symptoms. Overall, 9.4% of patients receiving risankizumab and 8.0% of patients in the placebo group experienced AEs possibly related to the drug. However, more patients in the placebo group experienced severe AEs (10.2%) than in the risankizumab group (2.5%). No significant cardiovascular events, active tuberculosis or severe hypersensitivity reactions were observed. Only 0.6% of risankizumab patients discontinued treatment due to AEs, compared to 3.7% in the placebo group [76].

Patients who responded to induction therapy were eligible for participation in the COMMAND maintenance study (NCT03398135) [77], while patients who did not respond were eligible for a further 12 weeks of induction therapy.

In the COMMAND study (NCT03398135), the role of risankizumab in the maintenance treatment of moderate to severe UC was further investigated [77]. Patients who responded to induction therapy received 180 mg, 360 mg risankizumab or placebo every 8 weeks for 52 weeks. Patients receiving 180 mg and 360 mg achieved significantly higher clinical remission rates (40.2% and 37.6%, respectively) compared to placebo (25.1%).

The overall rates of AEs and serious infections were similar among treatment groups. Serious events per 100 patient-years (E/100 PY) were lower in the risankizumab arms compared to placebo (risankizumab 180 mg: 5.9; risankizumab 360 mg: 6.3; placebo: 11.4), and serious AEs were also lower in the risankizumab arms (risankizumab 180 mg: 1.6; risankizumab 360 mg: 4.0; placebo: 8.0). No cases of active tuberculosis, anaphylaxis, severe hypersensitivity reactions or serious adverse cardiovascular events were reported in any of the treatment groups [77].

#### Risankizumab in CD

The role of risankizumab in the treatment of CD has been investigated in several studies, [74, 78–81] (Table 5), most notably in the phase 3 ADVANCE and MOTIVATE studies [80]. These randomized, double-blind, placebo-controlled studies involved adults with moderate to severe CD who had not responded adequately to one or more approved biologics or conventional therapy (ADVANCE) or biologics (MOTIVATE). Participants received either 600 mg or 1200 mg intravenous risankizumab or placebo at weeks 0, 4 and 8. The primary analysis included 850 participants from the ADVANCE study and 569 from the MOTIVATE study.

In the ADVANCE study, the clinical remission rate at week 12 according to CDAI was 45% with 600 mg and 42% with 1200 mg risankizumab, compared to 25% with placebo (*p* < 0.0001). The endoscopic response rates were also significantly higher in the risankizumab groups. The endoscopic response rate was 40% with risankizumab 600 mg and 32% with risankizumab 1200 mg compared to 12% with placebo (*p* < 0.0001). In the MOTIVATE study, clinical remission rates of 42% and 40% were also recorded for the respective doses compared to 20% with placebo (*p* < 0.0001). In addition, the endoscopic response rate was 29% with risankizumab 600 mg and 34% with risankizumab 1200 mg compared to 11% with placebo (*p* < 0.0001). In terms of safety, the rates of adverse events in the two treatment groups were comparable overall (ADVANCE: 56% in the risankizumab 600 mg group vs. 51% in the risankizumab 1200 mg group vs. 56% in the placebo group, MOTIVATE: 48% vs. 59% and 66%, respectively). In both studies, the most common AEs in the risankizumab groups were headache and nasopharyngitis. Three deaths occurred, during the induction phase, two in the placebo group (ADVANCE) and one in the risankizumab 1200 mg group (MOTIVATE) none of which were associated with the drug [79, 80].

The FORTIFY study, a phase 3 study, investigated the efficacy of risankizumab as maintenance therapy [81]. Participants who had responded to treatment in ADVANCE or MOTIVATE received a subcutaneous dose of 180 mg or 360 mg risankizumab every 8 weeks for 52 weeks. Clinical remission rates, as defined by the CDAI, at week 52 were 52% for the 360 mg dose and 55% for the 180 mg dose, both significantly higher than placebo (*p* = 0.0054 and *p* < 0–0001, respectively). Endoscopic response rates were 47% on risankizumab compared to 22% on placebo. In addition, higher rates of clinical CDAI remission and endoscopic response were observed at week 52 with a 180 mg dose of risankizumab compared to placebo (*p* = 0–0031 and *p* < 0–0001, respectively). Specifically, CDAI clinical remission was achieved in 55% with risankizumab 180 mg, and endoscopic response in 47%. The incidence of AEs was comparable in all groups (72% for risankizumab 180 vs. 72% for risankizumab 360 mg vs. 73% in the placebo group), with the most frequently reported AE in each treatment group being exacerbation of CD (11% for risankizumab 180 vs. 12% for risankizumab 360 mg vs. 17% in the placebo group), following by arthralgia (8% for risankizumab 180 vs. 9% for risankizumab 360 mg vs. 11% in the placebo group) and headache (5% for risankizumab 180 vs. 6% for risankizumab 360 mg vs. 6% in the placebo group) [81].

Real-world data from Belgium and tertiary reference centers confirmed these results. In particular, in the Belgian multicenter cohort study, 69 patients with CD were examined, most of whom had previously undergone at least four advanced therapies (85.5% with ≥ 4 different advanced therapies and 98.6% with ustekinumab, 14 with a stoma). All participants received three induction infusions of 600 mg risankizumab at weeks 0, 4 and 8, followed by a subcutaneous maintenance dose of 180 or 360 mg every 8 weeks, starting at week 12 [74].

Clinical remission was measured by an average daily stool frequency of ≤ 2.8 and a daily abdominal pain score of ≤ 1. Endoscopic response required a reduction of 50% or more from baseline. At week 24, 18.2% of patients without a stoma achieved steroid-free clinical remission, which increased to 27.3% by week 52. Half of the 32 patients with endoscopic data achieved an endoscopic response within 52 weeks, with similar remission rates in the patients with a stoma (steroid-free clinical remission rates of 14.3%). At a median follow-up of 68.3 weeks, 18.8% of patients discontinued risankizumab and 20.3% underwent bowel resection. The estimated surgery-free survival rate at week 52 was 75.2% and no new safety concerns were identified [74].

Again, 145 patients with CD were examined at a tertiary reference center [75]. The efficacy cohort included 80 patients with active luminal CD characterized by a Harvey-Bradshaw Index (HBI) of 5 or higher, or with active disease confirmed by imaging, ileocolonoscopy, or elevated fecal calprotectin levels. They received intravenous risankizumab (600 mg) at weeks 0, 4 and 8.

Most patients (61%) had undergone bowel resection in the past and only 8% did not respond to advanced therapies. HBI scores declined steadily throughout the induction period, dropping from 6 at baseline to 2 at week 12. Clinical remission rates gradually improved, reaching 70% at week 12. Three patients discontinued treatment before week 12 due to disease worsening. In the efficacy cohort, 36 patients (45%) had never received ustekinumab and 44 (55%) had previous experience with this drug. At week 12, 78% of ustekinumab-naïve patients and 64% of ustekinumab-experienced patients achieved clinical remission (*p* = 0.222). Steroid-free clinical remission was achieved by 75% of ustekinumab-naïve patients and 52% of ustekinumab-experienced patients (*p* = 0.041). Overall, 63% of patients achieved a steroid-free clinical remission. In the multivariate analysis, a history of bowel resection and a high baseline HBI reduced the likelihood of achieving steroid-free remission by week 12 (*p* = 0.005 for both). Safety data from 145 patients showed that 7.5% of patients experienced a disease exacerbation requiring steroid therapy, treatment modification or surgery. One patient discontinued treatment due to hypersensitivity after the first infusion. Other adverse events included fatigue, upper respiratory tract infections, joint pain and worsening of eczema [75].

Another multicenter, real-world study found that induction therapy with risankizumab in highly refractory patients with luminal Crohn's disease (CD) and multiple treatment failure, including ustekinumab, resulted in clinical response in approximately 75% of patients and steroid-free clinical remission in approximately 50% [82]. In addition, the ongoing APRISE study (NCT05841537) is collecting real-world post-marketing data on the efficacy and safety of risankizumab in the treatment of CD (Table 5).

#### Mirikizumab in UC

Two randomized, double-blind, placebo-controlled phase 3 trials of mirikizumab were conducted in adults with moderately to severely active ulcerative colitis (NCT03518086 and NCT03524092) [83]. In the induction trial, 1281 patients were randomized in a 3:1 ratio and received either mirikizumab (300 mg) or placebo intravenously every 4 weeks for 12 weeks. In the maintenance trial, 544 patients who had shown a positive response to induction therapy with mirikizumab were randomized in a 2:1 ratio to receive either mirikizumab 200 mg or placebo subcutaneously every 4 weeks for 40 weeks. Patients who did not respond during the induction trial were offered the option to receive mirikizumab as extended induction therapy during the initial 12 weeks of the maintenance trial. In both the induction trial (week 12) and the maintenance trial (week 52), the proportion of patients achieving clinical remission was significantly higher in the mirikizumab group than in the placebo group (24.2% vs. 13.3%, *p* < 0.001 and 49.9% vs. 25.1%, *p* < 0.001, respectively). Among the 1217 patients who received mirikizumab during the controlled and uncontrolled phases covering the open-label extension and maintenance phases of both trials, opportunistic infections occurred in 15 patients (including 6 cases of herpes zoster infection) and cancer was diagnosed in 8 patients (including 3 with colorectal cancer). In contrast, among patients receiving placebo in the induction trial, only one had a herpes zoster infection and none were diagnosed with cancer [83].

In addition, there are two ongoing studies evaluating the long-term efficacy and safety of mirikizumab in UC (NCT03519945) with a particular focus on the symptom of bowel urgency (NCT05767021) (Table 6). Preliminary data show that among patients who achieved clinical remission at week 52, the rate of maintenance of clinical remission at week 104 was 65.6%. In patients without prior biologic failure, the rate was 67.3%, while in patients with prior biologic failure, the rate was 61.7%. Among patients who were in clinical remission at week 52, 74% of them maintained symptomatic remission at week 104, and 64.3% were also in corticosteroid-free remission at week 104 [84].

**Table 6:** Mirikizumab in Ulcerative colitis (UC) and Crohn’s Disease (CD)

NCT number	Study acronym	Type of study	Aim	Study population	Primary outcome measures	Sponsor	Phases	Study status
NCT03518086 and NCT03524092	LUCENT-1 and LUCENT-2	Phase 3, Multicenter, Randomized, Double-Blind, Parallel, Placebo-Controlled Studies of Mirikizumab	To evaluate the safety and efficacy of Mirikizumab as induction therapy (LUCENT-1) or as maintenance therapy (LUCENT-2)	Patients With Moderately to Severely Active UC who have had an inadequate response to, loss of response, or intolerant to conventional or biologic therapy for UC (18–80 Years)	Percentage of Participants in Clinical Remission At Week 12 Percentage of Participants in Clinical Remission at Week 40 (at 52 weeks overall)	Eli Lilly and Company	Phase-3	ACTIVE, NOT RECRUITINGResults available 2024–03-15 (estimated) 2024-12-20 (estimated)
NCT03519945	LUCENT-3	A Phase 3, Multicenter, Open-Label Extension Study	To Evaluate the Long Term Efficacy and Safety of Mirikizumab	Patients With Moderately to Severely Active UC (18–80 Years)	Percentage of patients in clinical remission based on modified MS at week 52	Eli Lilly and Company	Phase-3	ACTIVE RECRUITING Completion Date (estimated) 2027-12-20
NCT05767021	LUCENT-URGE	A Multicenter, Phase 3b, Open-Label, Single-Arm Study to Investigate Bowel Urgency	To investigate bowel urgency in adults with moderately to severely active UC treated with Mirikizumab	Adults with moderately to severely active UC (18 Years and older)	Change from Baseline in Bowel Urgency Severity Urgency Numeric Rating Score	Eli Lilly and Company	Phase-3	ACTIVE RECRUITING Completion Date (estimated) 2025-03-31
NCT02891226	SERENITY	A Phase 2, Multicenter, Randomized, Parallel-Arm, Placebo-Controlled Study	To evaluate the safety and effectiveness of Mirikizumab	Adult participants with active CD as determined by the SES-CD, and reported stool frequency and abdominal pain (18–75 Years)	Percentage of Participants Achieving Endoscopic Response At Week 12	Eli Lilly and Company	Phase -2	COMPLETED (2021-02-05) with results
NCT03926130	VIVID-1	A Phase 3, Multicenter, Randomized, Double-Blind, Placebo- and Active- Controlled, Treat-Through Study	To Evaluate the Efficacy and Safety of Mirikizumab	Patients With Moderately to Severely Active CD as assessed by stool frequency, abdominal pain, and SES-CD (15–80 Years)	Percentage of Participants Achieving Clinical Response,as assessed by stool frequency, abdominal pain, and Patient Reported Outcome, at Week 12 and Clinical Remission (based on CDAI) and Endoscopic Response (based on SES-CD) At Week 52	Eli Lilly and Company	Phase-3	COMPLETED (2023-10-02) No results available
NCT04232553	VIVID-2	A Phase 3, Multicenter, Open-Label, Long-Term Extension Study	To Evaluate the Long-Term Efficacy and Safety of Mirikizumab	Patients With CD who have completed study NCT02891226 or study NCT03926130 (18 Years and older)	Percentage of Participants Achieving Endoscopic Response (based on SES-CD) and Clinical Remission (based on CDAI) At Week 52	Eli Lilly and Company	Phase-3	RECRUITING Completion Date (estimated) 2026-12-20

*MS* Mayo score, *SES-CD* Simple endoscopic score for Crohn's disease, *CDAI* Crohn's disease activity index, *FCP* Fecal calprotectin

#### Mirikizumab in CD

The efficacy and safety of mirikizumab in CD were investigated in a randomized phase 2 study [85]. In this trial, 191 patients were randomized (2:1:1:2) to receive placebo, 200, 600 or 1000 mg mirikizumab, administered intravenously (IV) every 4 weeks. Patients who received mirikizumab and achieved at least a 1-point improvement in Simple Endoscopic Score-CD at week 12 (rerandomized maintenance cohort) were re-randomized to continue their induction IV treatment (combined IV groups [IVC]) or to receive 300 mg of mirikizumab subcutaneously (SC) every 4 weeks. The non-randomized maintenance cohort included endoscopic non-improvers (1000 mg) and placebo patients (placebo/1000 mg) who received 1000 mg mirikizumab IV starting at week 12. The primary objective was to evaluate the superiority of mirikizumab over placebo in achieving an endoscopic response (50% reduction in Simple Endoscopic Score-CD from baseline) at week 12. At week 12, the endoscopic response was significantly higher at the predefined two-sided significance level of 0.1 for all mirikizumab groups compared to placebo (200 mg: 25.8%, 8/31, 95% confidence interval CI 10.4–41.2, *P* = 0.079; 600 mg: 37.5%, 12/32, 95% CI 20.7–54.3, *P* = 0.003; 1000 mg: 43.8%, 28/64, 95% CI 31.6–55.9, *P* < 0.001; placebo: 10.9%, 7/64, 95% CI 3.3–18.6). Endoscopic response at week 52 was 58.5% (24/41) and 58.7% (27/46) in the IV-C and SC groups, respectively. The incidence of AEs in the mirikizumab groups during the first 12 weeks was similar to that in the placebo (treatment-related AEs: 70.3% for placebo, 58.1% for mirikizumab 200 mg, 65.6% for mirikizumab 600 mg and 65.6% for mirikizumab 1000 mg). Until week 52, the incidence of treatment-related serious AEs was similar in all groups. The incidence of serious AE and discontinuation due to AE was higher in the non-randomized maintenance group than in the randomized maintenance group (13.6% and 10% vs. 0 and 3.4%; 11.9% and 10% vs. 2.4% and 2.2%, respectively) [85].

The two other studies on the use of mirikizumab in CD are VIVID-1 (NCT03926130) and the ongoing long-term extension VIVID-2 (NCT04232553) (Table 6). Notably, preliminary data show that mirikizumab was non-inferior to ustekinumab in clinical remission as assessed by CDAI (*p* = 0.113) [86].

### Guselkumab and brazikumab

These antibodies, which also target the p19 subunit, are currently under investigation. Preliminary data suggest a potential benefit in the treatment of IBD.

#### Guselkumab in CD

The potential role of guselkumab in moderate to severe CD was investigated in GALAXI-1, a double-blind, placebo-controlled phase 2 trial [87]. In this study, patients were randomized 1:1:1:1:1 to receive either intravenous guselkumab at 200 mg, 600 mg or 1200 mg at weeks 0, 4 and 8, intravenous ustekinumab at a dose of approximately 6 mg/kg at week 0 and 90 mg subcutaneously at week 8, or placebo. The primary endpoint was the change from baseline in the CDAI. Of the 309 patients studied, approximately 50% were refractory to prior biologic therapy. At week 12, a significantly greater reduction from baseline in CDAI was observed in each guselkumab group compared to placebo (least squares means: 200 mg: –160.4, 600 mg: –138.9, and 1200 mg: –144.9 versus placebo: –36.2; all, *P* < 0.05). Moreover, a significantly greater proportion of patients in each guselkumab group achieved clinical remission compared to placebo (CDAI < 150; 57.4%, 55.6% and 45.9% vs. 16.4%; all, *P* < 0.05). Rates of safety-related events were generally similar across treatment groups: in the 360 patients analyzed, a comparable proportion of patients experienced one or more AEs in all treatment groups by week 12 (placebo: 60.0%; guselkumab combined: 45.7%; and ustekinumab: 50.7%) [87].

After the efficacy of guselkumab as an induction therapy in moderate to severe CD was demonstrated, the role of guselkumab as a meta-drug therapy was investigated in a randomized, multicenter, double-blind phase 2 trial in adult patients [88]. In this study, 309 patients (112 biologics-naïve; 197 biologics-experienced) were randomly assigned to one of five treatment groups. Treatment regimens consisted of an intravenous induction phase followed by a subcutaneous maintenance phase beginning at week 12 in a treat-through design: from the guselkumab 200 to 100 mg group: 200 mg intravenously at weeks 0, 4 and 8, followed by 100 mg subcutaneously every 8 weeks (61 patients); from the guselkumab 600 to 200 mg group: 600 mg intravenously at weeks 0, 4 and 8, followed by 200 mg subcutaneously every 4 weeks (63 patients); from guselkumab 1200 to 200 mg group: 1200 mg intravenously at weeks 0, 4 and 8, followed by 200 mg subcutaneously every 4 weeks (61 patients); ustekinumab group: approximately 6 mg/kg intravenously at week 0, then 90 mg subcutaneously every 8 weeks (63 patients) and placebo group (61 patients): Placebo induction followed by either placebo maintenance (for those with clinical response according to CDAI at week 12) or crossover to ustekinumab (for those without CDAI clinical response at week 12). At week 48, the number of patients who achieved a clinical CDAI response (CDAI score < 150) was as follows: 39 (64%) in the guselkumab 200 → 100 mg group, 46 (73%) in the guselkumab 600 → 200 mg group, 35 (57%) in the guselkumab 1200 → 200 mg group and 37 (59%) in the ustekinumab group. The corresponding number of patients who achieved an endoscopic response (≥ 50% improvement in SES-CD or SES-CD score ≤ 2) was 27 (44%), 29 (46%), 27 (44%) and 19 (30%), respectively, and endoscopic remission (SES-CD score ≤ 2) was observed in 11 (18%), 11 (17%), 20 (33%) and four (6%) patients, respectively. In the placebo group, 15 patients were in clinical CDAI remission (either clinical CDAI remission or a decrease in CDAI score of ≥ 100 points from baseline) at week 12 and continued placebo treatment; of these, nine (60%) were in clinical remission at week 48. Forty-four patients in the placebo group were not in clinical CDAI remission at week 12 and switched to ustekinumab; of these, 26 (59%) were in clinical remission at week 48. Up to week 48, the frequencies of AEs in the safety population (*n* = 360) were as follows: 46 of 70 patients (66%) in the placebo group (464.9 events per 100 patient-years of follow-up), 163 of 220 patients (74%) in the three guselkumab groups combined (353.1 per 100 patient-years), and 60 of 71 patients (85%) in the ustekinumab group (350–7 per 100 patient-years). Among patients treated with guselkumab or ustekinumab, the most commonly reported infections through week 48 were nasopharyngitis (25 [11%] of 220 guselkumab recipients, 12 [11%] of 114 ustekinumab recipients) and upper respiratory tract infections (13 [6%] guselkumab recipients, eight [7%] ustekinumab recipients). After week 12, severe infections occurred in one patient who responded to placebo induction and in two patients treated with guselkumab. There were no cases of active tuberculosis, opportunistic infections or deaths [88].

Two other ongoing studies on the use of guselkumab in CD are GRAVITI (NCT05197049) and FUNZION CD (NCT05347095), which focus on fistulating, perianal CD (Table 7).

**Table 7:** Guselkumab in Crohn’s Disease (CD) and Ulcerative colitis (UC)

NCT number	Study acronym	Type of study	Aim	Study population	Primary outcome measures	Sponsor	Phases	Study status
NCT03466411	GALAXI-1 GALAXI-2 GALAXI-3	Program comprising Phase 2/3, Randomized, Double-blind, Placebo- and Active-controlled, Parallel-group, Multicenter studies	To evaluate the clinical efficacy (GALAXI 1), clinical and endoscopic efficacy (GALAXI 2 and GALAXI 3) and safety of Guselkumab	Participants with CD with inadequate response to conventional or to biologic therapy for CD (18 Years and older)	Phase 2: Change from Baseline in the CDAI Score. At Week 12 Phase 3: - Clinical Response (based on CDAI score) at Week 12 - Clinical Remission (based on CDAI score) at Week 48 - Endoscopic Response at Week 48	Janssen Research & Development, LLC	Phase-2, Phase-3	ACTIVE, NOT RECRUITING Completion Date (estimated) 2030-06-30
NCT05197049	GRAVITI	A Randomized, Double-blind, Placebo-controlled, Parallel-group, Multicenter Study	To evaluate the efficacy and safety of Guselkumab as Subcutaneous Induction Therapy	Participants with CD (18 Years and older)	Clinical Remission based on CDAI At Week 12 Endoscopic Response, based on change from baseline in SES-CD At Week 12	Janssen Research & Development, LLC	Phase-3	ACTIVE, NOT RECRUITING Completion Date (estimated) 2025-06-30
NCT05347095	FUZION CD	A Phase 3, Randomized, Placebo-controlled, Parallel-group, Multicenter Study to Evaluate the Efficacy and Safety of Guselkumab	To evaluate the clinical efficacy of Guselkumab in fistulizing, perianal CD and to assess the overall safety of Guselkumab	Participants With Fistulizing, Perianal CD (18 Years and older)	Percentage of Participants who Achieve Combined Fistula Remission at Week 24	Janssen-Cilag Ltd	Phase-3	ACTIVE, RECRUITING Completion Date (estimated) 2027-05-17
NCT04033445	QUASAR	A Phase 2b/3, Randomized, Double-blind, Placebo-controlled, Parallel-group, Multicenter Protocol	To evaluate the efficacy and safety of Guselkumab	Participants with moderately to severely active UC (18 Years and older)	- Induction Study 1: Clinical Response per modified MS. At Week 12 - Induction Study 2: Clinical Remission, per modified MS. At Week 12 - Maintenance Study: Clinical Remission, per modified MS. At Week 44	Janssen Research & Development, LLC	Phase-2, Phase-3	ACTIVE, NOT RECRUITING Completion Date (estimated) 2027-10-31

*SES-CD* Simple endoscopic score for Crohn's disease, *CDAI* Crohn's disease activity index, *MS* Mayo score, *FCP* Fecal calprotectin

#### Guselkumab in UC

The efficacy and safety of guselkumab as induction therapy in moderate to severe CU was investigated in QUASAR, a randomized, double-blind phase 2b trial [89]. This study involved 313 patients who had previously been treated with conventional or advanced therapy. Patients were randomly assigned to either placebo or guselkumab at a dose of 200 mg every 4 weeks or guselkumab at a dose of 400 mg every 4 weeks. At week 12 of the induction phase, the percentage of patients exhibiting a clinical response was 27.6% in the placebo group, 61.4% in the lower-dose guselkumab group and 60.7% in the higher-dose guselkumab group (*P* < 0.001). The safety results were largely consistent with previous studies in approved indications. The incidence of serious AEs was significantly lower at 1% in the guselkumab groups compared to 5.7% in the placebo group. Rare AEs requiring discontinuation of treatment were reported in 0.5% of patients in the guselkumab groups compared to 1.9% in the placebo group. Infection rates were comparable at 10.6% and 11.4%, respectively, with no serious infections occurring in the guselkumab groups compared to 1.9% in the placebo group. It is noteworthy that no deaths were recorded during the entire duration of the study [89].

#### Brazikumab in CD

The role of brazikumab (MEDI2070) in the treatment of moderate to severe CD was investigated in a double-blind, placebo-controlled phase 2a study [90]. In this study, 119 adults who had previously failed treatment with tumor necrosis factor antagonists were randomized to receive either 700 mg brazikumab or placebo intravenously at weeks 0 and 4, followed by subcutaneous doses of 210 mg starting at week 12. At week 8, a clinical response (defined as either a 100-point decrease in CDAI score from baseline or clinical remission with a CDAI < 150) was observed in 49.2% of patients treated with brazikumab compared to 26.7% in the placebo group, an absolute difference of 22.5%. At week 24, a clinical response was observed in 53.8% of patients who continued to receive open-label MEDI2070 and in 57.7% of patients who received placebo and then open-label MEDI2070 during the double-blind phase. Both groups had similar rates of AEs at week 12 (67.8% and 68.3%, respectively), with headache and nasopharyngitis being the most common [90].

Moreover, the safety of brazikumab was also investigated in an open-label phase of this study [91].

Patients who successfully completed the 12-week, received subcutaneous brazikumab every 4 weeks for 100 weeks. Of the 104 patients, 57 (54.8%) completed the entire treatment period, while 47 (45.2%) discontinued treatment, mainly due to lack of response (14.4%) or treatment-emergent AEs (TEAEs) (11.5%). Overall, TEAEs occurred in 44 (84.6%) of patients in the group that switched from placebo to brazikumab (placebo/brazikumab) and 43 (82.7%) in the group that continued with brazikumab (brazikumab/brazikumab), with mild to moderate infections being the most common (40.4% of patients in the placebo/brazikumab group and 50% in the brazikumab/brazikumab group).

No major adverse cardiac events, malignancies or deaths were reported during the study period [91].

The efficacy and safety of brazikumab in CD is also being investigated in the 52-week INTREPID study (NCT03759288) and its open-label extension (NCT03961815), but results are not yet available. (Table 8).

**Table 8:** Brazikumab in Crohn’s Disease (CD) and Ulcerative colitis (UC)

NCT number	Study acronym	Type of study	Aim	Study population	Primary outcome measures	Sponsor	Phases	Study status
NCT01714726	NA	Two-part Phase 2a study (12-week, double-blind, placebo-controlled, treatment period followed by a 100-week, open label, treatment period)	To Evaluate the Efficacy and Safety of MEDI2070	Subjects With Moderate to Severe CD Who Have Failed or Are Intolerant to Anti-tumor Necrosis Factor-alpha Therapy. (Adults:18-65 Years)	Clinical response (a CDAI decrease of 100 points from baseline or clinical remission [CDAI < 150]) at week 8	AstraZeneca	Phase-2	COMPLETED Results available
NCT02574637	NA	A Phase 2b Double-Blind, Multi-Dose, Placebo-Controlled Study to Evaluate the Efficacy and Safety of MEDI2070	To evaluate the efficacy and safety of brazikumab (MEDI2070) in	Participants with moderate to severe CD who have failed or are intolerant to anti-TNFα therapy (Adults: 18-80 Years)	Percentage of Participants With CDAI Remission at Week 8	AstraZeneca	Phase-2	COMPLETED (2018-01-29) Results available
NCT03759288	INTREPID	A 52-Week, Multicenter, Randomized, Double-blind, Placebo and Active-Controlled, Operationally Seamless Phase 2b/3, Parallel-group Study	To evaluate the safety and efficacy of brazikumab versus placebo (Stage I) and versus an active comparator (Stage 2)	Participants With Moderately to Severely Active CD (Adults: 18-80 Years)	Percentage of patients with CDAI remission at week 12. Percentage of patients with Endoscopic response (50% decrease from Baseline in SES-CD total score) at week 52. Percentage of patients in clinical remission	AstraZeneca	Phase-2	TERMINATED (2023-10-18)
NCT03961815	INTREPID OLE	Open-label Extension Study of Brazikumab in CD	To evaluate efficacy and safety of Brazikumab	Participants With Moderately to Severely Active CD (Adults: 18-80 Years)	Number and percentage of patients with reported AEs at week 70. Percentage of patients with potentially clinically significant changes in hematology, clinical chemistry, urinalysis at week 70. Percentage of patients with potentially clinically significant changes in systolic and diastolic blood pressure, temperature, respiratory rate and pulse rate at week 70. Percentage of patients with potentially clinically significant changes in 12-lead ECG recordings at week 70	AstraZeneca	Phase-3	TERMINATED (2023-09-19)
NCT03616821	EXPEDITION	A 54-Week, Multicenter, Randomized, Double-blind, Placebo-Controlled, Parallel-group Phase 2 Study	To evaluate the efficacy and safety of brazikumab	Patients with moderately to severely active UC Adults (18- 80 years)	Clinical remission defined as modified MS at week 10	AstraZeneca	Phase 2	TERMINATED (2023-10-23)
NCT04277546	EXPEDITION OLE	A Phase 2 Open-label, Long-term Extension Safety Study	To evaluate long-term safety and efficacy of brazikumab	Participants With Moderately to Severely Active UC Adults (18-80 Years)	Number and percentage of patients with reported AEs at week 70. Percentage of patients with potentially clinically significant changes in hematology, clinical chemistry, urinalysis at week 70. Percentage of patients with potentially clinically significant changes in systolic and diastolic blood pressure, and pulse rate at week 70. Percentage of patients with potentially clinically significant changes in full physical exams at week 70 Percentage of patients with potentially clinically significant changes in 12-lead ECG recordings at week 70	AstraZeneca	Phase 2	TERMINATED (2023-10-10)

*SES-CD* Simple endoscopic score for Crohn’s Disease, *CDAI* Crohn’s disease activity index, *MS* Mayo score*,* *FCP* Fecal calprotectin, *AEs* Adverse events

#### Brazikumab in UC

The role of brazikumab in moderate to severe UC was investigated in a 54-week, multicenter, randomized, double-blind, placebo-controlled, parallel-group phase 2 study (Expedition Lead-in- NCT03616821). In addition, the long-term efficacy and safety of this therapy in moderate to severe UC was analyzed in an open-label extension of the study (NCT04277546) (Table 8). The results of these studies are currently not available.

## Positioning novel treatments in the IBD armamentarium

In the treatment of CD and UC, the introduction and positioning of novel molecular therapies must be highly tailored, not only according to the primary disease activity but also considering patient-specific factors such as existing comorbidities, disease location, previous treatment history, patient preference, and safety profiles. The decision is highly personalized and based on a comprehensive assessment of the patient’s clinical history. For instance, ozanimod and etrasimod, which share the same mechanism of action, are administered orally, that is highly beneficial for enhancing patient adherence, especially in settings where infusion or injection therapies are less desirable. Importantly, despite clinical trials focusing on moderate to severe cases, clinical discussions suggest potential utility of S1P modulators in milder forms of the disease, though this broader application is not yet widely documented in the literature. Etrasimod, expected to be approved for use in patients from 16 years of age, may soon involve pediatric care, broadening its utility and improving accessibility for younger patients with IBD.

Anti-IL-23 agents, particularly appealing for patients who have only partially responded to other biologics, are crucial for maintenance therapy due to their favorable safety profile. For example, these agents are less likely to impact the immune system compared to broader immunosuppressants, making them particularly suitable for patients at higher risk of malignancy. They also offer the advantage of longer dosing intervals, which can significantly improve treatment adherence and quality of life. Specifically, Risankizumab has demonstrated significant efficacy in treating patients with moderate-to-severe CD who have not adequately responded to anti-TNF therapies. Preliminary results from the SEQUENCE trial (NCT04524611) highlight the superiority of risankizumab over ustekinumab, showcasing its effectiveness in achieving endoscopic remission and mucosal healing at both 24 and 48 weeks, positioning it as a preferred option for managing moderate-to-severe CD [92].

When determining the treatment sequencing, it is crucial to consider the previous response to therapy. For patients who have already been treated with biologics, particularly those with refractory disease, the introduction of newer agents such as ozanimod, etrasimod or anti-IL-23 should be considered as a step-up therapy. Furthermore, the combination of therapies, although requiring careful monitoring for additive immunosuppressive effects, could be used strategically to target different pathologic pathways simultaneously to improve overall disease control and remission rates. Further studies are expected to investigate the use of these drugs in sequence or in combination along with real-world data for use in specific clinical conditions involving comorbidities. This stratified approach will ensure a comprehensive treatment strategy that tailors therapeutic interventions to individual patient profiles and optimizes both efficacy and safety.

## Conclusion

Understanding the complex relationship between the immune system and gastrointestinal health is key to treating IBD. Recent advances in immunology have yielded promising therapeutic interventions that target specific signaling pathways. S1P modulators such as ozanimod and etrasimod reduce immune cell trafficking to curb inflammation, while IL-23 inhibitors disrupt a primary inflammatory driver, potentially enabling tailored, effective treatment.

Despite these innovations, potential side effects and limitations must be considered. S1P modulators may increase the risk of infection due to reduced immune surveillance [26, 44] and potentially reduce the efficacy of vaccines [93]. Similarly, although IL-23 inhibitors are designed to selectively modulate specific immune responses, concerns remain regarding long-term safety, particularly the risk of chronic immunosuppression [44]. Therefore, although these treatments offer significant benefits, their use must be carefully weighed against these risks.

**Fig. 2:**
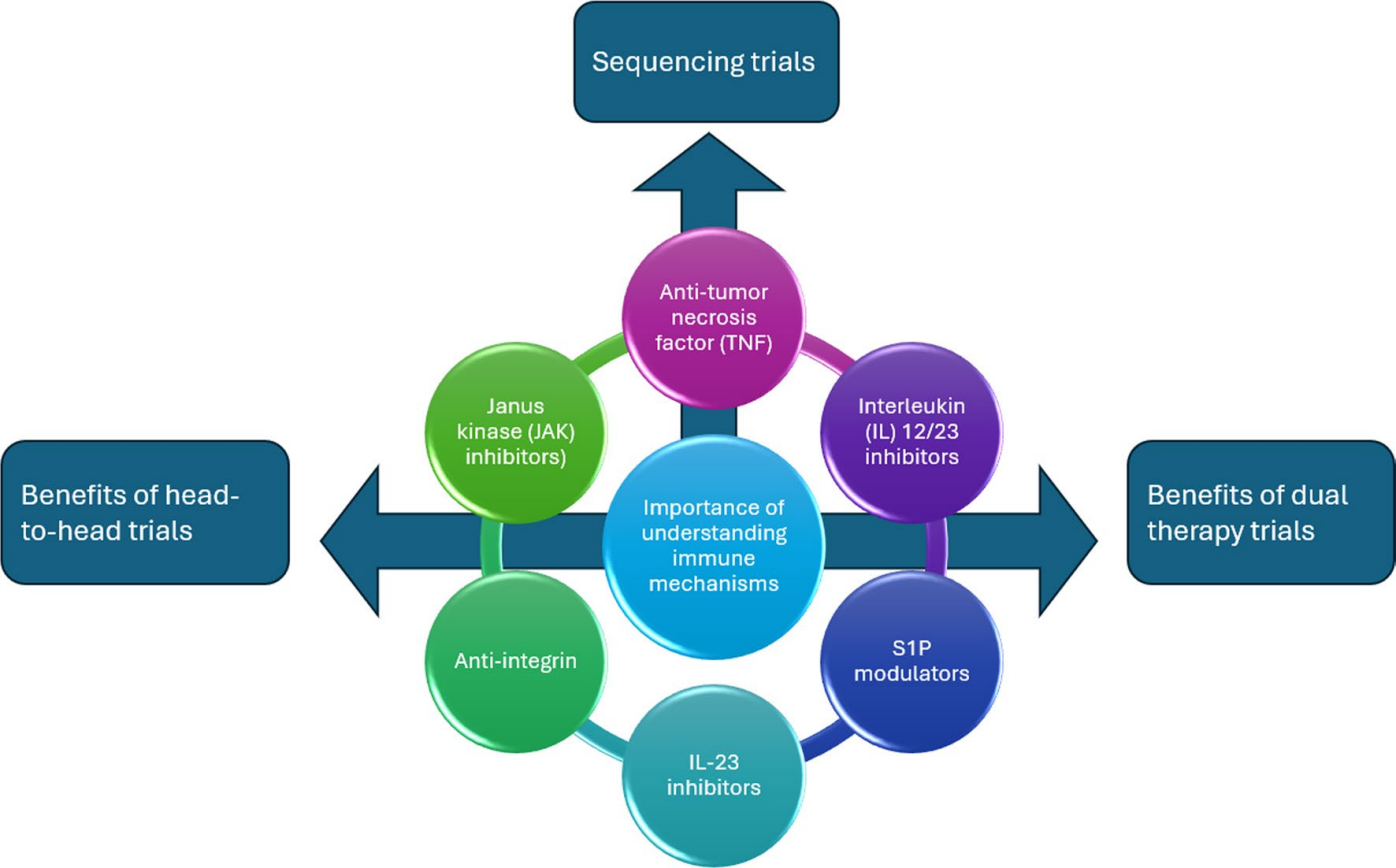
Combined strategies for optimizing inflammatory bowel disease (IBD) therapies

The evolving landscape of IBD treatment suggests that future advances will require head-to-head trials and exploration of the emerging concept of dual therapy. Ongoing trials such as the VEGA trial [94], the DUET-UC trial (NCT05242484) and the DUET-CD trial (NCT05242471) are examples of a shift towards exploring combination therapies to achieve better outcomes for IBD patients [95]. Indeed, combining biologic agents or targeting multiple pathways simultaneously can lead to synergistic effects that enable better disease control and remission 96. Moreover, head-to-head trials comparing different treatment modalities will be pivotal in determining the most effective interventions tailored to individual patient profiles (Fig. 2). These studies will provide crucial insights into different treatments' comparative efficacy and safety and guide physicians towards the most effective strategies tailored to each patient.

Deeper insight into the immune mechanisms of IBD will drive innovation and help improve patient outcomes and quality of life.
